# College students’ screening early warning factors in identification of suicide risk

**DOI:** 10.3389/fgene.2022.977007

**Published:** 2022-11-10

**Authors:** Ke Han, Lei Ji, Changfeng Chen, Binyin Hou, Decheng Ren, Fan Yuan, Liangjie Liu, Yan Bi, Zhenming Guo, Na Wu, Mofan Feng, Kai Su, Chenliu Wang, Fengping Yang, Xi Wu, Xingwang Li, Chuanxin Liu, Zhen Zuo, Rong Zhang, Zhenghui Yi, Yifeng Xu, Lin He, Yi Shi, Tao Yu, Guang He

**Affiliations:** ^1^ Bio-X Institutes, Key Laboratory for the Genetics of Developmental and Neuropsychiatric Disorders, Shanghai Jiao Tong University, Shanghai, China; ^2^ Shanghai Key Laboratory of Psychotic Disorders, and Brain Science and Technology Research Center, Shanghai Jiao Tong University, Shanghai, China; ^3^ School of Mental Health, Jining Medical University, Jining, Shandong, China; ^4^ Shanghai Center for Women and Children’s Health, Shanghai, China

**Keywords:** college students, suicide risk, early warning, masking effect, affiliation attribution

## Abstract

This study aimed to explore the main influencing factors of suicide risk among Chinese students and establish an early warning model to provide interventions for high-risk students. We conducted surveys of students in their first and third years from a cohort study at Jining Medical College. Logistic regression models were used to screen the early warning factors, and four machine learning models were used to establish early warning models. There were 8 factors related to suicide risk that were eventually obtained through screening, including age, having a rough father, and CES-D, OHQ, ASLEC-4, BFI-Neuroticism, BFI-Openness, and MMC-AF-C scores. A random forest model with SMOTE was adopted, and it verified that these 8 early warning signs, for suicide risk can effectively predict suicide risk within 2 years with an AUC score of 0.947. Among the factors, we constructed a model that indicated that different personality traits affected suicide risk by different paths. Moreover, the factors obtained by screening can be used to identify college students in the same year with a high risk of suicide, with an AUC score that reached 0.953. Based on this study, we suggested some interventions to prevent students going high suicide risk.

## Introduction

Suicide is a behavioral expression of serious psychological distress that causes serious public health concerns. Data from the World Health Organization (WHO) show that more than 700 000 people die due to suicide every year (WHO, 2022). For every person who dies due to suicide, there are many more people who attempt suicide (WHO, 2021). Globally, suicidal ideation (SI) and suicide attempt (SA) are more common in the younger population (!!! INVALID CITATION !!! (Eskin et al., 2005; Eskin et al., 2011)). Studies have shown that suicide is one of the main causes of death among adolescents and young adults, including among those in China (Benton et al., 2021).

The rates of suicide mortality vary among countries, and suicide behavior is influenced by ethnicity (Nock et al., 2008; Värnik, 2012). A study on American college students found that approximately 6.3% of college students had SI (Eisenberg et al., 2013). In a survey of suicide among college students in multiple countries, the proportion of Chinese college students who had SI was 22.9%, and the proportion with SA was 6.3% (Eskin et al., 2016). A study involving more than 8,000 Chinese colleges reported that the prevalence of SA was 6.8% (Shen et al., 2020). A meta-analysis involving 160339 Chinese college students calculated that the prevalence of SI was 10.72% (Li et al., 2014). Although the exact number varies among studies, the high frequency of suicide-related manifestations among Chinese college students should be a concern.

In a longitudinal study, it was shown that subjects who had SI during adolescence were twice as likely to have a Diagnostic and Statistical Manual of Mental Disorders (DSM) Axis I disorder and were nearly 12 times more likely to have attempted suicide by the age of 30 (Reinherz et al., 2006). It was also reported that these young patients have caused some pressure on local hospitals and medical systems (Eskin et al., 2016). Considering these facts, it is critical to pay attention to suicide in college students.

Many studies have focused on the risk factors for suicide to better understand suicide (Garlow et al., 2008; Breslin et al., 2020; Costanza et al., 2021). It has been reported that the strong risk factors for suicidal behavior (SB) in college students include psychological distress or depression (Garlow et al., 2008), low social support, affective dysregulation, alcohol use disorder (Arria et al., 2009), and depressogenic cognitive style (Hiramura et al., 2008). In addition, there are also reports indicating that adverse life events, family history, a history of sexual abuse, troubled relationships, impulsivity and difficulties with sexual identity can be risk factors for suicide (Nemeroff et al., 2001; Cooper et al., 2002; Arria et al., 2009; Costanza et al., 2021). The importance of the genetics of suicide was also emphasized in some studies, such as rs7989250, rs589046 and rs199633759, which suggesting the indispensable role of heritability (Otsuka et al., 2019; Erlangsen et al., 2020; Kimbrel et al., 2022; Mullins et al., 2022).

Of all the suicide studies, less attention has been given to early warning signs and identifying suicide in Chinese college students, and potentially important factors such as personality, happiness and attribution method have not been systematically investigated. Previous studies have mainly focused on SI and SA which may overlook some subjects who are at potential risk of suicide and the Suicidal Behaviors Questionnaire-Revised (SBQ-R) could help in this point, which is a well-validated approach to examine students’ suicide risk (Becker et al., 2018).

Therefore, in this work, we aimed to study the suicide situation of Chinese college students and identify the risk factors that affect suicide in Chinese college students, which, in addition to conventional factors, also include subjective well-being and attribution style, to better investigate this issue. This study also tried to establish a suitable early warning model and an identification model for suicide risk *via* a proper machine learning model.

## Materials and methods

### Subjects and data collection

Data in this study were collected from a cohort study of Jining Medical College in Shandong, China. The participants in this study were 3,630 Chinese college students from 28 provinces and 263 cities. The freshmen who enrolled in 2016 were selected to complete a basic demographic survey, family situation survey, and other relevant questionnaire surveys, which were used to investigate and evaluate the psychological status of the individuals. In their third year of school, we again conducted this data collection procedure and collected questionnaires about their suicide risk.

In both the baseline and follow-up groups, the exclusion criteria included a questionnaire completion time <600 s, feedback questions with unserious responses (efficacy, understanding, carefulness, significance), and K-means clustering analysis filtering. Furthermore, answers with inconsistencies regarding SI and SA were dropped.

All participants signed the informed consent form, and the study was appraised and approved by the Ethics Committee of Jining Medical University.

### Measurements

We used the questionnaire survey to collect information including basic demographic information, family economic and educational situations, mental illness situation of relatives, subjective well-being, life events and attributional causality (details are provided in Table 1 and Table 2). We then integrated these heterogeneous data to explore the factors that most influence college students’ suicide risk.

**TABLE 1:** Demographic description of the samples.

		Low risk of suicide	High risk of suicide	Statistic	*p*-value	Fisher p^a^
Age		21.568 ± 0.812	21.414 ± 0.729	2.184	**0.029**	
Weight		60.597 ± 16.154	60.793 ± 15.309	0.519	0.604	
Height		1.675 ± 0.0816	1.672 ± 0.0782	−0.139	0.89	
BMI		21.455 ± 4.942	21.643 ± 4.573	−0.437	0.662	
Gender				0.476	0.490	
	Female	631	43			
	Male	1,249	97			
Register				0.782	0.376	
	Urban	695	57			
	Rural household	1,185	83			
Sleep				27.861	**<0.001**	**<0.001**
	Extremely bad	15	2			
	Bad	97	16			
	Ordinary	789	79			
	Good	776	34			
	Very good	203	9			
Exercise				1.295	0.730	0.675
	<0.5 h	1,157	92			
	0.5–1 h	610	39			
	1–2 h	98	8			
	>2 h	15	1			
						
Father education				2.575	0.631	0.553
	Primary school and below	233	22			
	The junior middle school	758	48			
	Secondary or senior high School	518	40			
	College or university Degree	348	28			
	Graduate or above	23	2			
Mother education				1.979	0.740	0.665
	Primary school and below	468	37			
	The junior middle school	717	48			
	Secondary or senior high School	440	31			
	College or university Degree	242	23			
	Graduate or above	13	1			
Family income				0.776	0.679	0.723
	Below average	508	42			
	Average level	1,302	94			
	Above average	70	4			
Monthly living expense						
	Less than 500 yuan	144	3	7.970	0.093	0.058
	500–1,000 yuan	888	69			
	1,000–1,500 yuan	629	53			
	1,500–2000 yuan	152	8			
	More than 2000 yuan	67	7			
religious				4.360	0.037	0.058
	No	1830	132			
	Yes	50	8			
Father character				27.892	**<0.001**	**0.001**
	Serious	431	31			
	A bright and cheerful Disposition	596	43			
	Moderate	675	42			
	Rough	29	11			
	Reticence	149	13			
Mother character				20.354	**<0.001**	**0.001**
	Serious	109	17			
	A bright and cheerful Disposition	775	45			
	Moderate	954	70			
	Rough	22	6			
	Reticence	20	2			
Relatives suffer from mental illness						
	No	1844	131	12.188	**<0.001**	**0.003**
	Yes	36	9			
Variables	Estimate	Std. Error	OR	Lower 95% CI	Upper 95% CI	Z.value	*p*-value
Age	−0.25	0.12	0.78	0.62	0.97	−2.18	**0.029**
Sleep	−0.53	0.11	0.59	0.47	0.73	−4.79	**<0.001**
Father-Serious	−0.04	0.21	0.96	0.62	1.43	−0.21	0.832
Father -A bright and cheerful Disposition	−0.05	0.19	0.96	0.65	1.38	−0.24	0.808
Father-Moderate	−0.27	0.19	0.77	0.52	1.10	−1.40	0.160
Father-Rough	1.69	0.37	5.44	2.55	10.85	4.63	**<0.001**
Father-Reticence	0.17	0.30	1.19	0.63	2.08	0.57	0.568
Mother-Serious	0.81	0.28	2.25	1.27	3.77	2.92	**0.003**
Mother -A bright and cheerful Disposition	−0.39	0.19	0.68	0.46	0.97	−2.10	0.036
Mother-Moderate	−0.03	0.18	0.97	0.69	1.37	−0.17	0.865
Mother-Rough	1.33	0.47	3.78	1.37	8.94	2.84	**0.005**
Mother-Reticence	0.30	0.75	1.35	0.21	4.68	0.40	0.689
Relatives suffer from mental illness	1.26	0.38	3.52	1.56	7.15	3.28	**0.001**
PA	−0.12	0.02	0.89	0.86	0.92	−6.90	**<0.001**
NA	0.13	0.01	1.14	1.11	1.18	9.03	**<0.001**
CES-D	0.12	0.01	1.12	1.10	1.14	11.49	**<0.001**
SWLS	−0.16	0.02	0.85	0.82	0.88	−8.65	**<0.001**
OHQ	−0.21	0.02	0.81	0.77	0.84	−10.30	**<0.001**
ASLEC-1	0.13	0.02	1.14	1.10	1.19	7.05	**<0.001**
ASLEC-2	0.12	0.02	1.12	1.08	1.17	5.81	**<0.001**
ASLEC-3	0.04	0.01	1.04	1.02	1.06	3.46	**0.001**
ASLEC-4	0.10	0.02	1.10	1.06	1.15	4.36	**<0.001**
ASLEC-5	0.18	0.03	1.20	1.13	1.28	5.70	**<0.001**
ASLEC-6	0.11	0.02	1.12	1.07	1.17	5.00	**<0.001**
SSSUS-S	−0.17	0.02	0.84	0.81	0.88	−7.99	**<0.001**
SSSUS-O	−0.13	0.02	0.88	0.85	0.91	−7.58	**<0.001**
SSSUS-U	−0.12	0.02	0.88	0.86	0.91	−7.67	**<0.001**
BFI-Extraversion	−0.15	0.02	0.86	0.82	0.89	−7.52	**<0.001**
BFI-Agreeableness	−0.15	0.02	0.86	0.83	0.90	−6.92	**<0.001**
BFI-Conscientiousness	−0.15	0.02	0.86	0.83	0.90	−7.23	**<0.001**
BFI-Neuroticism	0.27	0.03	1.31	1.25	1.37	10.62	**<0.001**
BFI-Openness	−0.05	0.02	0.95	0.92	0.99	−2.43	**0.015**
GSES	−0.09	0.02	0.91	0.89	0.94	−5.54	**<0.001**
PALS-ASEC	−0.07	0.01	0.93	0.91	0.96	−5.13	**<0.001**
CPSE-SESRL	−0.10	0.02	0.91	0.88	0.94	−6.15	**<0.001**
MMC-AC-C	0.12	0.02	1.12	1.07	1.18	4.82	**<0.001**
MMC-AC-L	0.11	0.03	1.12	1.06	1.18	4.29	**<0.001**
MMC-AF-C	0.15	0.03	1.16	1.10	1.23	5.35	**<0.001**
MMC-AF-L	0.11	0.03	1.12	1.06	1.17	4.23	**<0.001**
UWES-S	−0.06	0.01	0.94	0.92	0.96	−6.56	**<0.001**

Note: Abbreviations: ASLEC-1 to ASLEC-6 represent six factors: interpersonal relationship, study pressure, punishment, sense of loss, healthy adaptation, and other factors; ASLEC, Adolescent Self-Rating Life Events Checklist; BFI, Big Five Inventory; CES-D, the Center for Epidemiological survey, depression scale; GSES, General Self-Efficacy Scale; CPSE-SESRL, Children’s Perceived Self-Efficacy Scales-Self-Efficacy for Self-Regulated Learning scale; MMC-AC-A, Multidimensional-Multiattributional Causality Achievement-Ability subscale; MMC-AC-E, Multidimensional-Multiattributional Causality Achievement-Effort subscale; MMC-AC-C, Multidimensional-Multiattributional Causality Achievement-Context subscale; MMC-AC-L, Multidimensional-Multiattributional Causality Achievement-Luck subscale; MMC-AF-A, Multidimensional-Multiattributional Causality Affiliation-Ability subscale; MMC-AF-E, Multidimensional-Multiattributional Causality Affiliation-Effort subscale; MMC-AF-C, Multidimensional-Multiattributional Causality Affiliation-Context subscale; MMC-AF-L, Multidimensional-Multiattributional Causality Affiliation-Luck subscale; NA, negative affect; OHQ, Oxford Happiness Questionnaire; PA, positive affect; PALS-ASEC, Patterns of Adaptive Learning Scales-Academic Self-Efficacy Scale; SWLS, Satisfaction with Life Scale; SSSUS-S, the Social Support Scale for University Students-Subjective support; SSSUS-O, the Social Support Scale for University Students-Objective support; SSSUS-SU, the Social Support Scale for University Students-Support Utilization; UWES-S, Utrecgt Work Engagement Scale-Student. Bold values indicate significant level (p<0.05)

^a^ Fisher’s exact test is used if necessary.

**TABLE 2:** Inter-group comparison of scale and questionnaire scores.

	Low risk of suicide	High risk of suicide	Statistic	*p*-value
PA	32.84 ± 5.09	29.67 ± 5.03	7.12	**<0.001**
NA	23.10 ± 6.27	28.41 ± 6.02	−9.71	**<0.001**
CES-D	13.77 ± 8.21	23.33 ± 8.62	−13.24	**<0.001**
SWLS	20.70 ± 4.99	16.72 ± 5.11	9.1	**<0.001**
OHQ	35.17 ± 5.43	29.74 ± 4.81	11.5	**<0.001**
ASLEC-1	11.82 ± 4.54	14.74 ± 4.36	−7.36	**<0.001**
ASLEC-2	13.03 ± 4.28	15.26 ± 4.26	−5.96	**<0.001**
ASLEC-3	12.85 ± 6.67	14.89 ± 6.58	−3.51	**<0.001**
ASLEC-4	6.20 ± 3.49	7.56 ± 3.41	−4.44	**<0.001**
ASLEC-5	6.94 ± 2.60	8.27 ± 2.52	−5.86	**<0.001**
ASLEC-6	7.85 ± 3.50	9.42 ± 3.52	−5.12	**<0.001**
SSSUS-S	20.03 ± 3.81	17.20 ± 3.94	8.45	**<0.001**
SSSUS-O	25.24 ± 4.35	22.16 ± 5.00	8.01	**<0.001**
SSSUS-U	23.94 ± 4.93	20.44 ± 5.08	8.09	**<0.001**
BFI-Extraversion	26.43 ± 4.53	23.33 ± 4.73	7.78	**<0.001**
BFI-Agreeableness	33.48 ± 4.45	30.72 ± 3.93	7.12	**<0.001**
BFI-Conscientiousness	29.85 ± 4.65	26.85 ± 4.50	7.39	**<0.001**
BFI-Neuroticism	22.47 ± 4.15	26.61 ± 3.86	−11.44	**<0.001**
BFI-Openness	34.25 ± 4.59	33.26 ± 4.64	2.44	**0.015**
GSES	35.89 ± 5.64	33.11 ± 5.69	5.62	**<0.001**
PALS-ASEC	43.23 ± 6.68	40.20 ± 6.18	5.2	**<0.001**
CPSE-SESRL	32.56 ± 5.66	29.46 ± 5.05	6.28	**<0.001**
MMC-AC-A	20.06 ± 2.81	20.41 ± 2.79	−1.4	0.163
MMC-AC-E	22.41 ± 2.85	21.93 ± 3.02	1.91	0.056
MMC-AC-C	17.07 ± 3.73	18.65 ± 3.10	−4.88	**<0.001**
MMC-AC-L	18.36 ± 3.51	19.68 ± 3.02	−4.31	**<0.001**
MMC-AF-A	19.05 ± 3.14	19.55 ± 3.34	−1.83	0.068
MMC-AF-E	20.29 ± 3.14	20.02 ± 3.35	0.97	0.33
MMC-AF-C	20.11 ± 3.00	21.55 ± 3.44	−5.43	**<0.001**
MMC-AF-L	17.97 ± 3.52	19.28 ± 3.35	−4.26	**<0.001**
UWES-S	77.60 ± 10.37	71.57 ± 8.30	6.72	**<0.001**

Note: Abbreviations: ASLEC-1 to ASLEC-6 represent six factors: interpersonal relationship, study pressure, punishment, sense of loss, healthy adaptation, and other factors; ASLEC, Adolescent Self-Rating Life Events Checklist; BFI, Big Five Inventory; CES-D, the Center for Epidemiological survey, depression scale; GSES, General Self-Efficacy Scale; CPSE-SESRL, Children’s Perceived Self-Efficacy Scales-Self-Efficacy for Self-Regulated Learning scale; MMC-AC-A, Multidimensional-Multiattributional Causality Achievement-Ability subscale; MMC-AC-E, Multidimensional-Multiattributional Causality Achievement-Effort subscale; MMC-AC-C, Multidimensional-Multiattributional Causality Achievement-Context subscale; MMC-AC-L, Multidimensional-Multiattributional Causality Achievement-Luck subscale; MMC-AF-A, Multidimensional-Multiattributional Causality Affiliation-Ability subscale; MMC-AF-E, Multidimensional-Multiattributional Causality Affiliation-Effort subscale; MMC-AF-C, Multidimensional-Multiattributional Causality Affiliation-Context subscale; MMC-AF-L, Multidimensional-Multiattributional Causality Affiliation-Luck subscale; NA, negative affect; OHQ, Oxford Happiness Questionnaire; PA, positive affect; PALS-ASEC, Patterns of Adaptive Learning Scales-Academic Self-Efficacy Scale; SWLS, Satisfaction with Life Scale; SSSUS-S, the Social Support Scale for University Students-Subjective support; SSSUS-O, the Social Support Scale for University Students-Objective support; SSSUS-SU, the Social Support Scale for University Students-Support Utilization; UWES-S, Utrecgt Work Engagement Scale-Student. Bold values indicate significant level (p<0.05)

Information on the social demographic questionnaire is shown in Table 1. Dichotomous variables were encoded using 0 and 1. Regarding the categorical variables, sleep quality was denoted as “1 = very bad”, “2 = bad”, “3 = normal”, “4 = good”, and “5 = very good”. Exercise amount was evaluated and recoded as “1 = < 0.5 h”, “2 = 0.5–11 h”, “3 = 1–2 h”, and “4 = > 2 h”. Parents’ education levels were recoded as “1 = primary school and below”, “2 = junior high school”, “3 = technical secondary school or high school”, “4 = college or university”, and “5 graduate school and above”. Parental character traits were encoded used five variables with 0 and 1. The five traits were coded with five variables, with 1 as having the trait and 0 as not having the trait. The five traits were serious, bright and cheerful disposition, moderate, rough and reticence. Living expenses were evaluated and recoded as “1 = < 500 yuan”, “2 = 500-1,000 yuan”, “3 = 1,000-1,500 yuan”, “4 = 1,500-2,000 yuan”, and “5 = > 2,000 yuan”. Whether immediate family members (parents, siblings, grandparents) had a mental illness was represented by 1 for illness and 0 for no illness.

The questionnaires included the Positive and Negative Affect Scale (PANAS), Center for Epidemiological Survey Depression scale (CES-D) (Li and Hicks, 2010), Oxford Happiness Questionnaire (OHQ) (Hills and Argyle, 2002), Satisfaction with Life Scale (SWLS) (Diener et al., 1985), Adolescent Self-Rating Life Events Checklist (ASLEC) (Li et al., 2020), Social Support Scale for University Students (SSSUS) (Ye and Dai, 2008), Utrecht Work Engagement Scale-Student (UWES-S), General Self-Efficacy Scale (GSES) (Luszczynska et al., 2005), Learning Scales-Academic Self-Efficacy Scale (PALS-ASEC) (Dever and Kim, 2016), Big Five Inventory (BFI), Multidimensional-Multi-attributional Causality Scale (MMCS) (Lefcourt, 1981), Patterns of Adaptive Children’s Perceived Self-Efficacy Scales-Self-Efficacy for Self-Regulated Learning scale (CPSE-SESRL) (Pastorelli et al., 2001) and Suicidal Behaviors Questionnaire-Revised (SBQ-R) (Osman et al., 2001). Moreover, four feedback questions and time elapsed were designed to select reliable data.

### Data analysis methods

Quantitative data were described by means (Xs) and standard deviations (SDs). Chi-square and t tests were used to compare the differences between the case group and the control group. Univariate and multivariable logistic regression (LR) models were used to establish the warning model of suicide risk. Principal component analysis (PCA) was used to further simplify the exact important factors. Mediation analysis was used to test the associations between factors. The synthetic minority oversampling technique (SMOTE) was used to address the class imbalance issue (Blagus and Lusa, 2013; Ryu et al., 2019). LR, linear support vector machine (SVM), Gaussian kernel SVM, and random forest (RF) models were used to evaluate the prediction results. For model development, 10-fold cross-validation was used to avoid overfitting and to increase the generalization of the model. In 10-fold cross-validation, data in the training set were partitioned into 10 equally sized folds, and each fold was used once as a validation set, while the other 9 folds were used for training. The area under the receiver operating characteristic (AUROC) curve was adopted to further evaluate the predictive characteristics of the acquired warning model. Statistical analysis software, including SPSS 22.0 and R studio, was used to analyze the data.

## Results

A total of 3,630 students completed the online questionnaires, and 2020 students were ultimately included in our study (Extended Data Figure 1). The surveyed students completed the SBQ-R in their third year of school and were divided into the high-risk group (total score of 7 or greater) and the low-risk group (total score of less than 7), which is generally used (Osman et al., 2001; Becker et al., 2018; Dizon et al., 2022).

**FIGURE 1:**
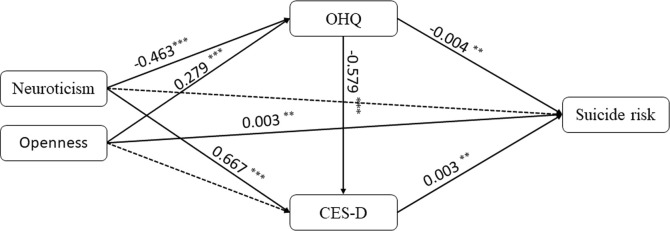
Path diagram for the mediation analysis modeling of the BFI-Neuroticism, BFI-Openness, OHQ, CES-D, and suicide risk. BFI, Big Five Inventory; CES-D, the Center for Epidemiological survey, depression scale; OHQ, Oxford Happiness Questionnaire.

We first compared the baseline variables of the two groups of students, as shown in Table 1. There were no significant differences (*p* > 0.05) between the two groups in some variables, including sex, weight, height, BMI, register, exercise status, father’s education level, mother’s education level, family economic condition, and family living expenses. However, we found that the average age of the high-risk group was significantly younger than that of the low-risk group (*p* = 0.029). The low-risk group showed a poor sleep status compared to the high-risk group (*p* < 0.001). There were statistically significant differences in three other variables: father’s character traits (*p* < 0.001), mother’s personality traits (*p* = 0.001), and relatives suffering from mental illness (*p* = 0.003). In total, two groups of major confounding variables were controlled, and they could be used to conduct on baseline data comparisons between the groups.

We found that there were significant differences between the low-risk group and the high-risk group in the scores (or subscale scores) of negative affect (NA), positive affect (PA), and the CES-D, OHQ, SWLS, ASLEC, SSSUS, BFI, GSES, PALS-ASEC, CPSE-SESRL, MMCS, and UWES-S (Table 2).

To simplify the early warning signs, we then used univariate logistic regression on all significantly different factors between the two groups for further factor screening, and a total of 34 factors were found to be significantly related to the suicide risk of college students (Extended Data Table 1). The 34 significant factors in univariate logistic regression were then included in the multivariable logistic regression analysis. The logistic regression (LR) stepwise multivariable logistic regression analysis method was applied to select the early warning predictors of suicide risk. According to different inclusion criteria (Sle) and exclusion criteria (Sls), the likelihood ratio forward entry method was adopted to carry out stepwise multivariate logistic regression analysis. Table 3 demonstrates the results of optimal multivariable LR. The final 8 early warning signs, were age, having a rough father, and the CES-D, OHQ, ASLEC-4, BFI-Neuroticism, BFI-Openness, and MMC-AF-C scores.

**TABLE 3:** Multivariate logistic regression for the suicide risk on selected variables.

Variables	Estimate	Std. Error	Z.value	*p*-value	
Age	−0.27	0.13	−1.99	0.047	*
Father-Rough	1.00	0.47	2.15	0.032	*
Mother -A bright and cheerful Disposition	0.60	0.32	1.90	0.058	.
Relatives suffer from mental illness	0.71	0.45	1.57	0.117	
CES-D	0.06	0.02	3.77	0.000	***
SWLS	−0.05	0.02	−1.92	0.055	.
OHQ	−0.11	0.03	−3.57	0.000	***
ASLEC-4	0.07	0.03	2.19	0.028	*
ASLEC-6	−0.07	0.04	−1.84	0.066	.
BFI-Neuroticism	0.12	0.03	3.81	0.000	***
BFI-Openness	0.08	0.03	3.08	0.002	**
MMC-AF-C	0.10	0.04	2.81	0.005	**
UWES-S	−0.02	0.01	−1.52	0.129	

Note: Abbreviations: CES-D, the Center for Epidemiological survey, depression scale; SWLS, Satisfaction with Life Scale; OHQ, Oxford Happiness Questionnaire; ASLEC, Adolescent Self-Rating Life Events Checklist; BFI, Big Five Inventory; MMC-AF-C, Multidimensional-Multiattributional Causality Affiliation-Context subscale; UWES-S, Utrecgt Work Engagement Scale-Student. ASLEC-4 and 6 represents: sense of loss

PCA revealed that the first four components had 70.7% variance of the 8 risk factors, and it is clearer to identify the exact important factors of suicide risk. The first factor mainly reflected the personality and subjective feeling dimension, the second factor indicated the age dimension, and the third and fourth factors both identified the mixed dimensions of having a father with a rough character, age, and sense of loss.

We noted that the first component included personality and subjective feeling information and then verified the association between the factors. We hypothesized that the OHQ and CES-D scores would be affected by neuroticism and openness and that other confounders would not be affected. The structural equation model was used to test the relationship among the BFI-Neuroticism, BFI-Openness, OHQ, and CES-D scores and suicide risk, while the other confounders were controlled. The indices indicated that the model fit (*p* < 0.001, CFI = 0.968, RMSEA = 0.068, SRMR = 0.027). The results showed that neuroticism could indirectly affect suicide risk according to the OHQ and CES-D scores, while no significant direct path existed (Figure 1). Openness could affect suicide risk both directly and indirectly. Different personalities affected suicide risk by different paths.

Next, we tested the effectiveness of the warning model for the 8 factors associated with suicide risk. The LR model, SVM (linear) model, SVM (Gaussian) model, and RF model were used to verify the prediction effectiveness under tenfold cross-validation. As indicated in Table 4, the accuracy, sensitivity, specificity, positive predictive value (PPV), and negative predictive value (NPV) for all four methods were over 76%, 67%, 85%, 82%, and 72%, respectively. The AUC in the LR, SVM (linear), SVM (radial), and RF models reached 0.770, 0.767, 0.870, and 0.947, respectively. We further assessed the predictive effect of the model with the first three components. The indices for the 4 models we tested decreased by varying degrees. The RF model still showed excellent performance, with the AUC reaching 0.925 (Table 4).

**TABLE 4:** Evaluation of models in early warning and suicide prediction and identification.

		Sensitivity	Specificity	PPV	NPV	Accuracy	AUC
Early warning	LR	0.689	0.852	0.823	0.733	0.771	0.770
	SVM (linear)	0.673	0.863	0.831	0.726	0.768	0.768
	SVM (radial)	0.842	0.900	0.894	0.851	0.871	0.871
	RF	0.917	0.978	0.976	0.922	0.947	0.947
Predict and identification	LR	0.795	0.856	0.846	0.807	0.825	0.825
	SVM (linear)	0.807	0.854	0.846	0.816	0.830	0.830
	SVM (radial)	0.891	0.895	0.895	0.892	0.893	0.893
	RF	0.935	0.972	0.970	0.937	0.953	0.953
PCA components for early warning	LR	0.617	0.666	0.648	0.636	0.641	0.641
	SVM (linear)	0.644	0.657	0.652	0.650	0.651	0.651
	SVM (radial)	0.818	0.696	0.728	0.793	0.757	0.757
	RF	0.930	0.920	0.921	0.930	0.925	0.925

Note: 750 Abbreviations: LR, logistic regression; RF, random forest; SVM, support vector machine; NPV, negative predictive value; PPV, positive predictive value; 751 AUC, area under receiver operating characteristic curve.

Considering that the suicide questionnaire has a certain influence on the minds of college students, we hoped to identify the suicide risk of college students by screening early warning signs, to avoid the psychological implications of suicide on college students and the subsequent adverse effects caused by such psychological implications. The four prediction models were again employed for 7 factors, and the results are shown in Table 4. The best identification effect was achieved by using the RF model, with an AUC of 0.953, and the accuracy, sensitivity, specificity, PPV, and NPV reached 95.3%, 93.5%, 97.1%, 97.0%, and 93.7%, respectively. The AUC value of the other three models also exceeded 0.82, which indicated that the 8 factors were capable of identifying suicide risk in college students.

## Discussion

We collected information on comprehensive factors that were conjectured to be associated with suicide risk and identified 8 important warning signs that can effectively predict suicide risk in college students. Some of the factors were consistent with previous studies, and some new points are worth noting (Figure 1).

**FIGURE 2:**
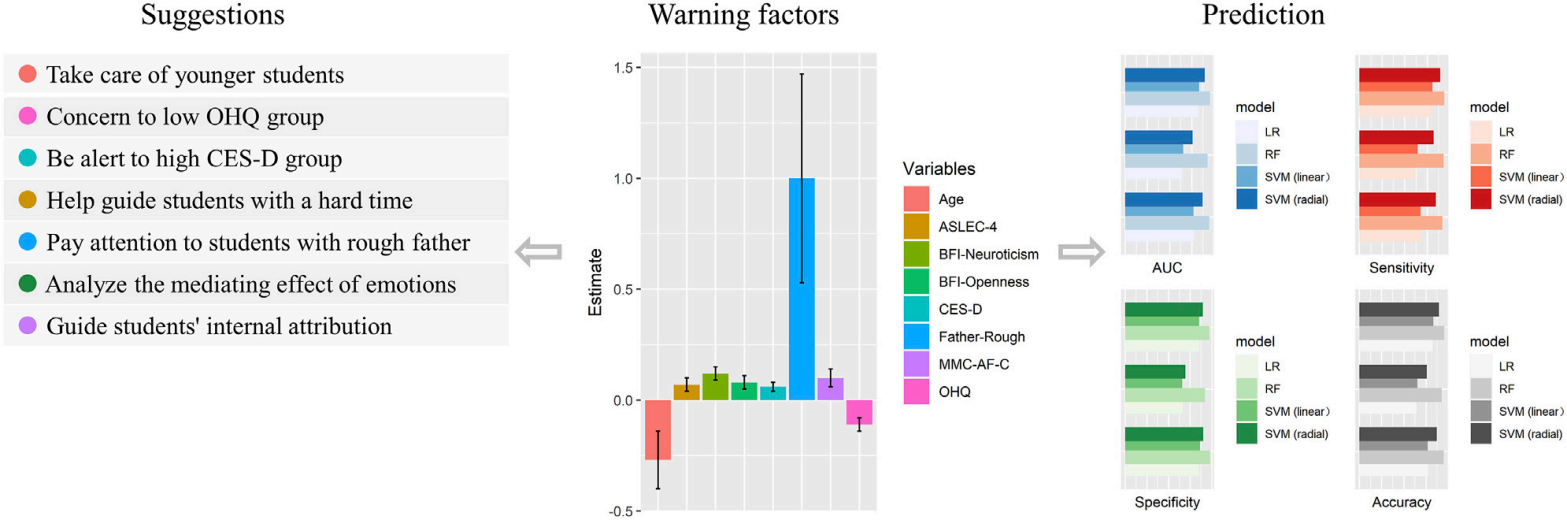
Overall schematic diagram of the warning factors, suggestions and model performance. CES–D, the Center for Epidemiological survey, depression scale; OHQ, Oxford Happiness Questionnaire; ASLEC, Adolescent Self–Rating Life Events Checklist; BFI, Big Five Inventory; MMC–AF–C, Multidimensional–Multiattributional Causality Affiliation–Context subscale; LR, logistic regression; RF, random forest; SVM, support vector machine; AUC, area under receiver operating characteristic curve. ASLEC–4 represents: sense of loss factor.

Our results showed that age was a protective factor against suicide risk among college students. Older students had a low risk of suicide. Studies have reported that older age was associated with less neuroticism and openness, which could be one of the reasons why suicide risk was lower in older students (Carciofo et al., 2016). Some studies divided people into various groups by age, while our study divided students using a cross-section (Ivey-Stephenson et al., 2022; Paul and Fancourt, 2022). The age difference between students in the low- and high-risk groups of suicide risk was significant but not much, which indicated that people should take more care of younger students in the same grade. The influence of family members has also been considered by many existing researchers (Svob et al., 2018; Kim, 2019). In this study, we discovered that between the low suicide risk group and the high suicide risk group, there was a significant difference in parental personality traits. Follow-up regression analysis confirmed that having a father with a rough personality would have a significant impact on the suicide risk of college students. Studies have reported that parental relationships and parental company have a certain influence on the SI of teenagers and college students (Saffer et al., 2015; Fu et al., 2017). Our research emphasizes the importance of parental personality traits in the process of getting along with their children. All the results indicated that the impact of parents on suicide risk is substantial, especially for college students, as they are in the transition stage from adolescence to young adulthood and can therefore be greatly influenced by their parents Figure 2.

The OHQ score was associated with suicide risk and is related to subjective well-being (SWB). SWB assessments are generally composed of multiple measuring scales, including the SWLS, PANAS, and OHQ (Hou et al., 2020). All three aspects of SWB were significantly associated with suicide risk in the univariate logistic regression results. Evidence has shown that SWB is negatively correlated with depression and that depression is always assciated with suicide. We found that SWB was important in early warning signs of suicide risk, and only the OHQ score was left in the final model. This study revealed that higher happiness levels, as a protective factor, were reflected in the suicide risk assessment. This is also consistent with the conclusions of other studies (Hsu et al., 2019; Hsu et al., 2020). The importance of the OHQ score indicates that not only are students with disease symptoms a focus group, but students with low happiness should also be given attention in school.

There are many studies on suicide clarifying the relationship between depression and SA and SI (Konick and Gutierrez, 2005; Garlow et al., 2008; Lewis et al., 2014). In our research, the mental state of depression was also found to be linked to the risk of suicide. A higher depressive mood can be an early warning sign of college students’ suicide risk, which is also in line with reports on other ethnic groups (AbdElmageed and Mohammed Hussein, 2022; Mullins et al., 2022; Qi and Li, 2022). The loss factor of the ASLEC described the negative life events of the loss of relatives, friends, and property over the last 6 months. Such negative life events increased the risk of suicide. Some studies reported that other factors of the ASLEC were significantly associated with suicide (or suicide-related symptoms) (Shao et al., 2021).

It was noted in this study that the neuroticism and openness of college students were also strong early warning signs for suicide risk. The results were different among different groups in other studies (Heisel et al., 2006; Bluml et al., 2013; Jo et al., 2021). People with a neurotic personality are generally considered to have difficulty handling emotions such as anxiety, hostility, depression, self-awareness, impulsivity, and vulnerability. For college students, their emotions might be unstable and uncontrollable, and college students with a neurotic personality may be more likely to have SI or SA because they have more difficulty in controlling their emotions. People with an openness personality might be open to new experiences, which might lead to a light decision to attempt suicide. College students with such personalities should be given special attention.

Openness, as a strong early warning sign of suicide risk in college students, also differed among different groups. A study of individuals with posttraumatic stress disorder (PTSD) showed that openness predicted lower suicide attempts (Yoo et al., 2018). Another interesting result in this study was that the PTSD group with much higher suicidality had higher scores in openness and neuroticism compared to the control group, while the logistic regressions showed that openness predicted lower SA and neuroticism with no significant result (Yoo et al., 2018). Our results showed a similar result: neuroticism scores were higher in the high-risk group, and openness scores were lower. Logistic regression showed that both neuroticism and openness predicted higher suicide risk. Further PCA showed that openness and the OHQ score had the opposite effect on suicide risk in multivariable logistic regression.

Based on these results, we verified that there was a masking effect between suicide risk and openness, mediated by OHQ and CES-D scores. This result indicated that among different groups, human personality traits played a different role in predicting suicide risk and could be affected by some confounding factors. The masking effect has been less discussed in other studies. This result suggested that the classification of the individuals involved in the study should be given attention.

Another point of concern is that attribution to the interpersonal context can lead to a greater risk of suicide, which means that if a college student attributes their problems or the difficulties encountered in interpersonal relationships to extraversion and attributes these occurrences to environmental factors beyond their control, they would be prone to a greater risk of suicide. We believe that this finding is meaningful for college students. Researchers have paid extensive attention to the role of interpersonal factors in suicide and interpersonal factors related to personality traits (Maxwell, 2005; Van Orden et al., 2010; Baertschi et al., 2017; Baertschi et al., 2018). Our results are useful for further understanding the attribution component of interpersonal relationships. Lectures on the attribution method may be of great help in protecting college students from suicide risk, and instructors are advised to remind students to become accustomed to looking for problems related to their own perspectives.

In this study, based on the 8 early warning signs, the students’ risk of suicide 2 years after completing the survey can be effectively predicted. In the RF model, the AUC value reached 0.947, which means that we established an effective early warning model to divide students into two groups: the low-risk and high-risk groups. This can benefit schools in taking preventative guidance measures for students at risk of suicide, helping them deal with psychological pressure and avoiding the further aggravation of suicide risk in appropriate ways.

We noted that among college students, the high-risk group was commonly unbalanced, with a small percentage. We verified that the SMOTE technique is an effective and valuable method used in suicide prediction, which has been less used in previous studies.

The models using the first three components of PCA showed poor performance in three models: the LR (AUC = 0.641), linear-SVM (0.651), and radial-SMV (0.757) models. However, the RF model provided a valuable result (AUC = 0.925), which indicated that the three components had given enough information to predict suicide risk and that researchers should better use and explore them.

Furthermore, we tested the identification effect of suicide risk in the same year based on the 8 factors. It was believed that it was beneficial to use the SBQ-R in the survey of suicide risk, and it can comprehensively investigate the situation related to suicide among students (Becker et al., 2018). The questionnaire methodology may, however, exert a certain influence on college students because it directly involves negative implications such as suicide itself. Therefore, we suggest using the 8 factors selected in this study to identify the suicide risk of college students instead of the SBQ-R, unless necessary. Models based on the 8 factors demonstrated effective discriminatory power. The AUC values of the four models all exceeded 0.82, and the AUC value of the RF model reached 0.953. This will assist schools in identifying whether college students are at high risk of suicide. Based on some studies, we note that the fear of the suicide survey’s implication for college students might not be entirely founded (Baertschi et al., 2019; Rudd and Bryan, 2022). Talking about suicidality does not encourage suicidality; in contrast, talking about it openly allows an individual to be able to express himself or herself without the fear of being judged. We should continue to pay attention to this effect among Chinese college students.

Our study also suggested a potential link for some genetic level explanations. A number of association studies on suicide have confirmed that some SNPS are loci that affect suicide (Otsuka et al., 2019; Erlangsen et al., 2020; Kimbrel et al., 2022). However, this attempt to explain the genetic factors affecting suicide from a single phenotype is insufficient. Our study suggests that some individual symptoms linked with suicide might help profile the genetic risk of suicide. It is reported that hedonic wellbeing was associated with some loci of FSHR on chromosome 2 and TRIM26 on chromosome 6 (Jamshidi et al., 2022). The rs3131073 was associated with some related conditions including positive-affect, wellbeing spectrum and depression (Howard et al., 2019; Jamshidi et al., 2022). The neuroticism has also identified positive correlative with anxiety disorder and MDD, while revealing a significant genetic overlap between depression and neuroticism (Kendler et al., 2006; Navrady et al., 2018; Forstner et al., 2021). To synthesize the characteristics associated with suicide, a comprehensive consideration of its genetic predisposition may lead to a deeper understanding of the genetic dimension of suicide.

This study also has certain limitations. The research subjects came from one college in China, and the results obtained need to be verified by a wider range of university student research. Additionally, the lack of suicide risk data in the students’ first year makes it difficult to verify the performance of identification in the first year. In this study, we note the importance of false alarms, which means that some students were not defined as being in the high-risk group for suicide but were alarmed by our system. The reason false alarms exist is that the data collected could not give a full view of high-suicide-risk students. False alarms remind us to include more aim indicators in data collection, such as impulsivity, near-infrared spectroscopy (NIRS), electroencephalogram (EEG), and some genetic indices (Lutz et al., 2017; Hirose et al., 2018; Costanza et al., 2021; Iznak et al., 2021; Koenig et al., 2021). Lifetime SA was significantly associated with both higher impulsivity and higher aggression (Costanza et al., 2021). In bipolar disorder patients, current suicide risk was noted to be significantly and positively associated with delayed activation timing of the NIRS signal in the prefrontal region (Hirose et al., 2018). EEG coherence was higher in the nonsuicidal self-injury (NSSI) and SA subgroups than in the NSSI subgroup, especially in the frontal–central–parietal regions. Some metabolic parameters and hormones are associated with suicide behaviors, which should be included in subsequent studies (O'Connor et al., 2020; Zhou et al., 2021). Thyroid stimulating hormone (TSH) could help to differentiate suicide attempters from nonsuicide attempters (Zhou et al., 2021). Cortisol was observed to be significantly associated with suicide attempts, while people younger than 40 years had a positive association, and those older than 40 years had a negative association with suicide attempts (O'Connor et al., 2020). Genome-wide significant SNPs of suicide were also needed to be noticed, such as rs589046, rs199633759, and rs77378519 (Otsuka et al., 2019; Erlangsen et al., 2020; Kimbrel et al., 2022).

In conclusion, we established a concise early suicide warning model and provided a simplified version of the suicide risk identification approach for college students’ suicide risk (Figure 1). The main factors in these models suggested that we should pay attention not only to strong internal and external factors but also to some predisposing factors that are not particularly valued by disease diagnosis, especially in the warning period of up to 2 years. We advocate for some suggestions, including improving students’ happiness, reducing their stress responses to life events, and guiding them to make positive attributions. We look forward to additional research examining the actual effects of these interventions on suicide risk among college students.

## Data Availability

The original contributions presented in the study are included in the article/Supplementary Material, further inquiries can be directed to the corresponding authors.

## Supplementary material

SUPPLEMENTARY FIGURE S1 Study flowchart.
