# Supplementary figures and images for: College students’ screening early warning factors in identification of suicide risk
Source: Front Genet. 2022 Nov 10;13:977007. doi: 10.3389/fgene.2022.977007 (PMC9710625; doi:10.3389/fgene.2022.977007)

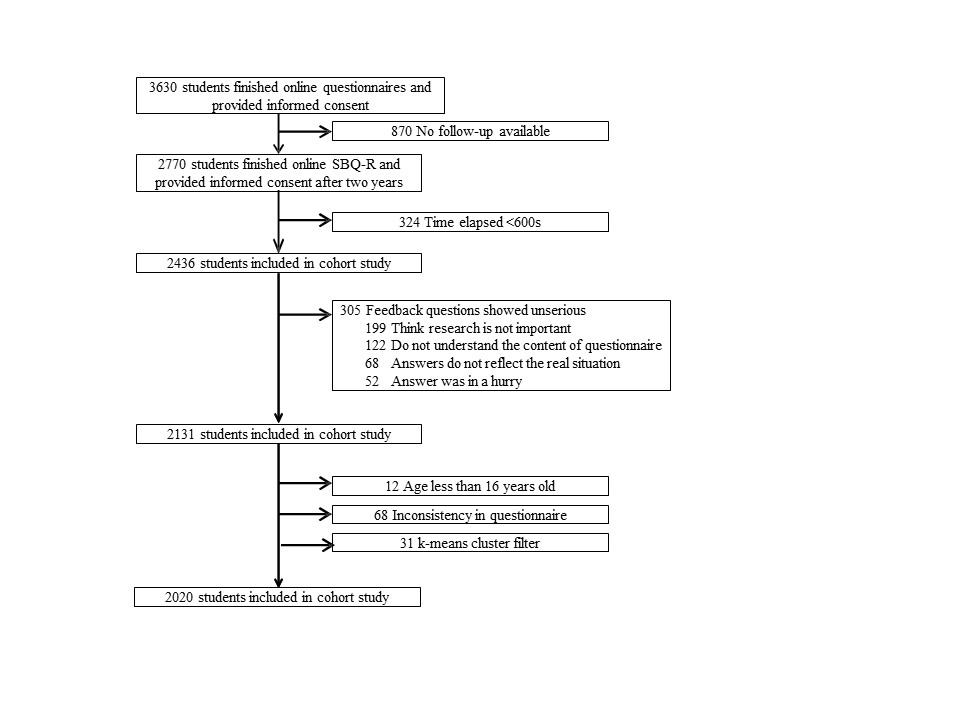

Supplement: Supplementary file 1 [file Image1.jpg]
